# Innate Immune Evasion by Human Respiratory Syncytial Virus

**DOI:** 10.3389/fmicb.2022.865592

**Published:** 2022-03-04

**Authors:** Yan Ouyang, Hongqun Liao, Yan Hu, Kaiyuan Luo, Shaowen Hu, Huifang Zhu

**Affiliations:** ^1^Neonatal/Pediatric Intensive Care Unit, Children's Medical Center, First Affiliated Hospital of Gannan Medical University, Ganzhou, China; ^2^Ganzhou Key Laboratory of Immunotherapeutic Drugs Developing for Childhood Leukemia, Ganzhou, China; ^3^Department of Gynecology, The Third Affiliated Hospital of Kunming Medical University, Kunming, China; ^4^Basic Medical College of Gannan Medical University, Ganzhou, China; ^5^Institute of Children's Medical, First Affiliated Hospital of Gannan Medical University, Ganzhou, China

**Keywords:** respiratory syncytial virus, innate immune response, PRRs, RLRs, TLRs, NLRs

## Abstract

Respiratory syncytial virus (RSV) is the leading cause of severe respiratory infection in young children. Nearly all individuals become infected in their early childhood, and reinfections with RSV are common throughout life. Primary infection with RSV is usually involved in the symptom of bronchiolitis and pneumonia in the lower respiratory tract, which accounts for over 3 million hospitalizations and approximately 66,000 deaths annually worldwide. Despite the widespread prevalence and high morbidity and lethality rates of diseases caused by RSV infection, there is currently no licensed RSV vaccine. During RSV infection, innate immunity plays the first line of defense to suppress RSV infection and replication. However, RSV has evolved multiple mechanisms to evade the host’s innate immune responses to gain a window of opportunity for efficient viral replication. This review discusses the comprehensive interaction between RSV infection and the host antiviral innate immunity and updates recent findings on how RSV modulates the host innate immune response for survival, which may provide novel insights to find potent drug targets and vaccines against RSV.

## Introduction

**Figure 1:**
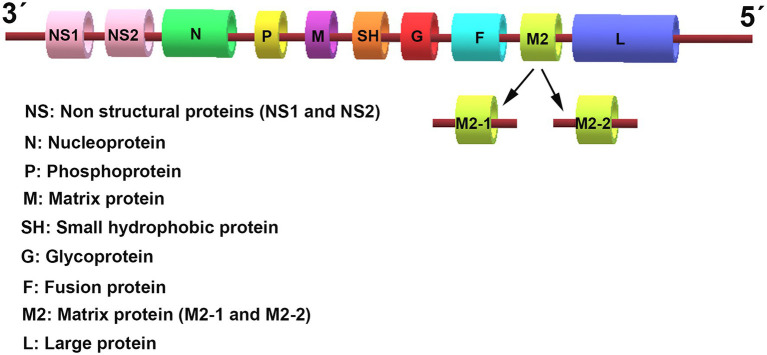
The genomic organization of respiratory syncytial virus (RSV). The RSV genome is approximately 15.2 kb in size and encodes 10 genes that transcribe 11 proteins. The genome sequence from 3′ to 5′ successively encodes the non-structural (NS) proteins 1 and 2 (NS1/2), the nucleocapsid (N), the phosphoprotein (P), the matrix (M) protein, the small hydrophobic (SH) protein, the attachment (G) protein, the fusion (F) glycoproteins, the M2 gene-encoded two distinct matrix proteins M2-1 and M2-2, and the large polymerase (L) protein.

Respiratory syncytial virus (RSV) is an enveloped virus belonging to the *Pneumovirus* genus in the *Paramyxoviridae* family. It contains a single-stranded, negative-sense RNA genome, which is 15.2 kb in size and comprises 10 genes that encode 11 viral proteins (Figure 1). These include two major surface proteins, the glycoprotein (G) and the gusion protein (F), and an envelope protein, the small hydrophobic (SH) protein. In addition, the matrix (M) proteins, the M2 gene-encoded two distinct matrix proteins M2-1 and M2-2, the nucleoprotein (N), the phosphoprotein (P), and the RNA polymerase large nucleoprotein (L), are located in the nucleocapsid. Two other non-structural (NS) proteins, NS1 and NS2, are characterized by their immunomodulatory functions. The airway epithelium is the primary target of RSV infection by binding viral G protein to cell-surface glycosaminoglycans. After attaching to epithelial cells, the viral surface F protein will mediate host cell membrane fusion to facilitate RSV entry.

**Figure 2:**
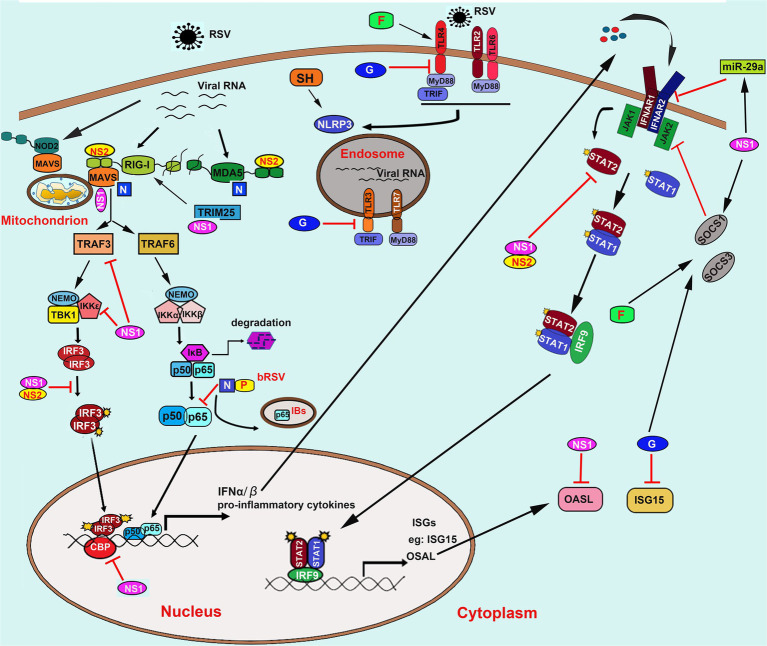
Innate recognition of RSV and its immune evasion mechanisms. RSV is recognized by TLRs including TLR2/6, TLR4, TLR3, and TLR7; RLRs including RIG-I and MDA5; NLRs including NOD2 and NLRP3. NS1/2 mainly mediates the innate immune evasion mechanisms caused by RSV converged in interfering with the RLRs signaling pathway by direct binding to RIG-I, MDA5, adaptor proteins MAVS, and TRIM25. Besides, NS1/2 also induces the decrease of TRAF3, IKKε, and blocks the phosphorylation of IRF3 and its association to transcriptional coactivator CBP. Moreover, NS1/2 also prevents the JAK/STAT signaling by promoting the degradation of STAT2 and OASL and inhibits the IFNAR synthesis *via* inducing the expression of miR-29a. Besides, NS1 can also induce the expression of SOCS1 in a RIG-I and TLR3-independent pathway, which further inhibits the phosphorylation of STAT2. The N protein binds to MDA5 and MAVS to inhibit their activation. The bovine RSV (bRSV) N protein can also cooperate with P protein to promote the formation of IBs-like structures to capture the p65 subunit of NF-κB, which further prevents its translocation to the nucleus. On the one hand, the soluble G protein blocks the TLR3/4-mediated IFN-β production. On the other hand, it also impedes the ISG15 expression. The G protein and F protein induce both SOCS1 and SOCS3, which subsequently interferes with the JAK–STAT pathway to block the IFN-I response. Abbreviation: RSV, respiratory syncytial virus; TLRs, toll-like receptors; RIG-I, retinoic acid-inducible gene I; MDA5, melanoma differentiation associated gene 5; NOD2, nucleotide-binding oligomerization domain 2; NLRP3, the NOD, leucine-rich repeat (LRR)-containing protein 3; NS1/2, non-structural (NS) protein 1 or 2; MAVS, mitochondrial antiviral-signaling protein; TRIM25, tripartite motif-containing protein 25; TRAF3/6, tumor necrosis factor (TNF) receptor-associated factor (TRAF) adapter protein 3 or 6; NF-κB, nuclear factor kappa-B; IκB, inhibitor of NF-κB; IKKε, inhibitor of IκB kinase ε; IRF3, interferon regulatory factor 3; JAK, Janus kinase; STAT, signal transducer and activation of transcription; OASL, 2′-5’-Oligoadenylate synthetases-like protein; IFNAR, IFN-α receptors; SOCS1/3, suppressor of cytokine signaling protein 1 or 3; N; nucleoprotein; P, phosphoprotein; G, glycoprotein; IBs, inclusion bodies; IFN-α/β, interferon α or β; ISG15, IFN-stimulated gene 15; CBP, cAMP (cyclic adenosine monophosphate) response element-binding protein (CREB)-binding protein.

Meanwhile, airway epithelial cells are also the first site for the activation of host innate immune responses against RSV infection through a wide variety of pattern recognition receptors (PRRs), including the toll-like receptors (TLRs) and the cytoplasmic retinoic acid-inducible gene (RIG)-I like receptors (RLRs). On the one hand, recognition of pathogen-associated molecular patterns (PAMPs) results in activation of antiviral type I interferon (IFN-I) response, which restricts virus replication and transmission; on the other hand, RSV has evolved several mechanisms to evade the host immune response and promote virus replication through interactions between RSV proteins and immune components. In this review, we discuss the interactions between RSV proteins and host factors that modulate the immune response and the implications of these interactions for the course of an RSV infection (Figure 2).

## Innate Immune Response Against RSV Infection

RSV infection is the leading cause of bronchiolitis and pneumonia in children. Although the symptoms in most patients would spontaneously relieve within a week, approximately 1 to 3% of infants with RSV infection require hospitalization for supportive care, which is responsible for approximately 50% of all types of pneumonia in infancy. It is proposed that reinfections with RSV are common throughout an individual’s lifespan, and the early exposure to RSV infection in life can lead to an increased susceptibility to suffering from recurrent allergic wheezing and asthma. The first infection usually evokes inadequate immunological memory, and reinfection may exacerbate inflammatory response in the respiratory tract promoting airway damage during virus clearance. In that case, on the one hand, RSV infection in the respiratory tract induces innate immune responses by the host to facilitate the efficient clearance of the virus; on the other hand, the exacerbated host immune response during RSV infection may also partly be responsible for lung pathology, characterized by immune cell infiltration to the lungs. Therefore, the host immune response also contributes to respiratory diseases following RSV infection, except for the immune protection role.

The host’s innate immunity is powerful in the first-line defense of viral infection. Multiple elements of the innate immune system, including diverse innate cell types, various PRRs, and a large array of cytokines and chemokines, contribute to the control of RSV infection. Generally, innate cellular immunity against virus infection is primarily mediated by IFN-I, which then exerts the pleiotropic effects by inducing a variety of IFN-stimulated genes (ISGs). Unique viral proteins/genomes and replication products from RSV are natural PAMPs to evoke host innate immune response through PRRs. To date, three major classes of PRRs: TLRs, RLRs, and NOD-like receptors (NLRs) are demonstrated to be involved in the detection of RSV, leading to the induction of cytokines, chemokines, and IFN-I, which subsequently facilitate the clearance of the virus.

### RSV Infection Triggers TLRs Pathway

TLRs are type I transmembrane receptor proteins composed of an extracellular domain containing multiple leucine-rich repeats, a transmembrane region, and a cytoplasmic tail containing the conserved TIR domain. The TLR family members typically recognize evolutionarily conserved structures of many pathogens, such as bacterial lipopolysaccharide, bacterial and viral nucleic acids, peptidoglycans, lipoproteins. Ten different TLRs (TLR1-10) have been identified in humans. They are largely divided into two subgroups depending on their cellular localization and respective ligands. One group is composed of TLR1 ~ 6, which are expressed on cell surfaces and recognize microbial membrane components such as lipoproteins and proteins; the other group is composed of TLR3, TLR7, TLR8, and TLR9, which are expressed in endosomes and recognize microbial nucleic acids.

TLRs are constitutively expressed on epithelial cells lining the skin and the respiratory, endothelial cells, and immune cells. The activation of TLRs signaling pathways leads to antiviral cytokines that support viral clearance. Several TLRs have been reported to be involved in RSV recognition. For example, TLR2, TLR3, and TLR4 on epithelial cells have been implicated in inducing cytokines and chemokines upon RSV infection (Rudd et al., 2005; Murawski et al., 2009). Leukocytes expressed TLR2, TLR3, TLR4, TLR6, and TLR7 can interact with RSV and promote immune responses following infection. Alveolar macrophages are the primary source of IFN-I in RSV-infected mice during the early periods of infection (Goritzka et al., 2015), but the IFN-I production by macrophages and dendritic cells (DCs) from wild-type (WT) and TLR1, -2, -4, -6, and - 7 knock-out (KO) mice is very similar following RSV infection, indicating that TLRs are essential for IFN-I production in this. While in another study, mice plasmacytoid dendritic cells (pDCs) were reported to induce IFN-I production through TLR7-MyD88 signaling pathway, but not the RLR-MAVS (mitochondrial antiviral-signaling protein) pathway *in vivo* (Kim et al., 2019). TLR2, which is expressed on immune cells and tissues, generally forms heterodimers with TLR1 or TLR6 to activate innate immunity. Genetic analysis of TLR-KO BALB/c mice demonstrated that TLR2/6 signaling, but not TLR2/1 signaling, is involved in early cytokine and chemokine production in response to RSV, while the induction of IFN-I was independent on TLR2 signaling (Murawski et al., 2009). TLR3 can detect double-stranded RNA (dsRNA) during viral infection. It is reported that TLR3 also contributes to cytokine production in human lung cells infected with RSV, independent of the IFN-I signaling (Rudd et al., 2005). On airway epithelial cells, the TLR4/CD14 complex is the main extracellular receptor recognizing RSV through interacting with viral F protein to activate the nuclear factor kappa-B (NF-κB)-mediated cytokine response, including the secretion of IL-6, IL-8, and IL-10 (Kurt-Jones et al., 2000; Haynes et al., 2001; Haeberle et al., 2002). Similarly, in murine peritoneal macrophages, F protein was also reported to induce IL-6 secretion dependent on the TLR4/CD4 signaling (Haeberle et al., 2002). In addition, RSV infection also upregulates TLR4 expression on airway epithelial cells in mice and peripheral blood monocytes and upper airways from infants with RSV bronchiolitis (Gagro et al., 2004). However, some other studies did not find a role for purified F protein in stimulating TLR4-dependent activation of NF-κB (Marr and Turvey, 2012). Therefore, the role of TLR4 in response to RSV infection is currently debated.

### RSV Infection Triggers the RLRs Pathway

The RLRs family comprises RIG-I and MDA5, widely expressed in most cell types, such as fibroblasts, epithelial cells, macrophages, and DCs. They are cytosolic RNA helicases/ATPases that possess two N-terminal caspase activation and recruitment (CARD) domains, a central DExD/H-box helicase domain and a C-terminal RNA-binding domain (CTD; Rehwinkel and Gack, 2020). They can recognize cytosolic pathogenic-derived ssRNA and short dsRNA to activate the downstream signaling pathway by the interaction between the CARD domain and the adaptor protein MAVS, which subsequently leads to the recruitment of members of the TNF (tumor necrosis factor) receptor-associated factor (TRAF) adapter proteins, mainly including TRAF3 and TRAF6 to promote the activation of interferon regulatory factor 3 or 7 (IRF3/7) and NF-κB, respectively (Seth et al., 2005). These activated transcription factors then translocate from the cytoplasm into the nucleus, ultimately inducing the production of IFN-I and the inflammatory genes. RIG-I senses 5′-triphosphate blunted ends in the presence of short ss/dsRNA (Cui et al., 2008; Jiang et al., 2011), while MDA5 predominantly recognizes long dsRNA without sequence specificity (Wu et al., 2013).

Sasai and colleagues first reported the involvement of RLRs in RSV infection in 2006 (Sasai et al., 2006). Their study demonstrated that RIG-I is essential for IFN-β induction in HeLa cells, dependent on RSV replication. RIG-I’s critical role in recognizing RSV was also shown in airway epithelial cells (Liu et al., 2007). It was demonstrated that the expression of RIG-I and MDA5 were upregulated, but only RIG-I could specifically bind to RSV RNA (RSV A2 strain) in human bronchiolar carcinoma cell line A549, which was confirmed by immunoprecipitation assay. Besides, the knockdown of RIG-I by siRNA approach could significantly block the activation of NF-κB and IRF3 and the production of IFN-β (Liu et al., 2007). Moreover, data from another literature also indicated that both IFN-α/β and IFN-λ (IFN-III) were induced by RSV infection in A549 cells in RIG-I-dependent pathway (Okabayashi et al., 2011). In RSV-infected hTERT-NECs, which is one type of immortalized human nasal epithelial cells (NECs) through transfection with the human telomerase reverse transcriptase gene, suppression of RIG-I expression but not MDA5 by short interfering RNA (siRNA) technology significantly reduced IFN-λ1 production, indicating that RIG-I but not MDA5 contributed to the main role in inducing the expression of IFN-III. However, studies from another group showed that both RIG-I and MDA5 could co-localize with RSV genomic RNA (RSV A2 strain) in the small inclusion bodies (IBs) in A549 cells by immunofluorescence assay (Lifland et al., 2012). A clinical study observed a significant positive correlation between RSV viral load and RIG-1 mRNA levels in these RSV-infected infants with bronchiolitis (Scagnolari et al., 2009). Also, in human primary PBMCs isolated from healthy volunteers, the expression of RIG-I was upregulated following RSV infection (Vissers et al., 2012). These studies demonstrate that RIG-I seems to been particularly important for host to defense against RSV infection. Similar results were also obtained in an *in vivo* infectious model (Demoor et al., 2012). The MAVS (the adaptor protein for RIG-I and MDA5) deficient mice produced reduced amount of IFN-I infection, as well as other pro-inflammatory cytokines, including interleukin-6 (IL-6), tumor necrosis factor-α (TNF-α), monocyte chemoattractant protein-1 (MCP-1), and IL-1β in response to RSV (Demoor et al., 2012).

### RSV Infection Triggers the NLRs Pathway

The NLR family of PRRs is intracellular cytoplasmic sensors that play a crucial role in the innate immune response against invading pathogens. There are 22 identified NLRs in humans and at least 33 NLRs in mice. Structurally, they share a central NOD (nucleotide-binding and oligomerization domain) domain, N-terminal effector domain, and C-terminal leucine-rich repeats (LRRs). Based on the different functions of the N-terminal effector domain, NLRs can be divided into four subfamilies: the acidic transactivation domain (NLRA), the baculoviral inhibitory repeat-like domain (NLRB), the caspase activation and recruitment domain (NLRC), and the pyrin domain (NLRP). They are widely involved in various intracellular activities, including inflammasome assembly, signaling transduction, transcription activation, and autophagy.

NOD2, a member of the NLRC subfamily, was recently demonstrated to be involved in recognizing ssRNA virus by activating the IFN-I-mediated innate antiviral responses. It is reported that RSV infection could rapidly increase NOD2 expression (within 2 h) after RSV and plays a critical role in the induction of IFN-β in 293 cells and PBMCs in a MAVS-dependent manner (Sabbah et al., 2009; Vissers et al., 2012). Mechanically, NOD2 could translocate to the mitochondria and interact with MAVS to activate IRF3 and NF-κB, resulting in the induction of IFN-I (Sabbah et al., 2009). Similar results were obtained from the mice model *in vivo*. It has been shown that, compared with the WT mice, the susceptibility of RSV and lung disease were significantly enhanced in NOD2-deficient mice. Moreover, the activation of IRF3 and production of IFN-I were also reduced in NOD2-deficient mice (Sabbah et al., 2009). These data indicate that NOD2 functions as an important PRR for RSV recognition and plays a critical role in the host’s innate immune defense against RSV infection.

NLRP3 [the nucleotide-binding oligomerization domain (NOD), leucine-rich repeat (LRR)-containing protein 3], another member of the NLR family, plays a crucial role in the innate immune response and inflammation by recruiting proCASP1 to the inflammasome, which ultimately leads to the proteolytic activation of the pro-inflammatory cytokines interleukin-1β (IL-1β) and interleukin-18. Recent evidence indicates that NLRP3 is also reported to be activated following RSV infection in mouse bone marrow-derived macrophages. The TLR2/Myd88 signaling pathway was demonstrated to be essential for the activation of NF-κB and subsequent expression of pro-IL-1β and NLRP3 caused by RSV infection (Segovia et al., 2012). Moreover, evidence from another research demonstrates that RSV appears to stimulate the inflammasome activation through both the TLR4 signaling and SH protein on its own. Upon the infection of RSV (strain A2) in primary human lung epithelial cells, the knocking down of TLR4, but not TLR3, TLR7, TLR8, or RIG-I results in a reduced expression of pro-IL-1β and IL-1β. In this process, TLR4 may act as a sensor for RSV recognition to trigger inflammasome activation during virus invasion.

Further study demonstrated that the RSV mutants lacking the viroporin SH could not trigger inflammasome activation. It was observed that during RSV infection, the SH protein accumulated in lipid rafts of the Golgi apparatus, which further enhances membrane permeability and promotes the entry of small molecules into cells; meanwhile, interestingly, NLRP3 was also shown to accumulate in the Golgi apparatus. It was inferred that the functions of SH viroporin may facilitate the trafficking of NLRP3 from the cytoplasm to the Golgi, which is essential for the inflammasome activation (Triantafilou et al., 2013).

However, the NLRP3-mediated excessive inflammation seemed to be responsible for lung tissue damage (Shen et al., 2018). Therefore, NLRP3 was suggested as a potential therapeutic target to protect lung disease from RSV infection.

## Evasion of Innate Immune Response by RSV

The interaction between host innate immunity and RSV is a long-term tug of war. On the one hand, the RSV infection evokes the production of IFN-I and numerous downstream ISGs to limit viral replication; on the other hand, RSV has evolved specialized strategies to modulate and suppress the IFN-I response to gain a window of opportunity for efficient virus replication and setting-up of the infection. Human epithelial cells, including A549, Hep-2, and HEK293 infected with either RSV A2 strain or clinical isolates, failed to produce IFN-α, suggesting that RSV could block the ongoing IFN-I signaling. However, understanding the innate immune evasion mechanisms employed by RSV is helpful for the design of target therapy. This review will focus on the implicated strategies for RSV to antagonize the host antiviral immune response.

### RSV Impedes the RLRs Signaling Pathway

Numerous groups have highlighted the role of NS proteins in suppressing innate immune signaling. It was indicated that the infection of recombinant human RSV with single or double deletions of NS genes (ΔNS1, ΔNS2, and ΔNS1/2) in A549 cells produce higher levels of IFN-I compared to WT virus (Spann et al., 2004). Moreover, in human macrophage and epithelial cells, the infection of RSV virus with both NS1 and NS2 genes deficient evokes higher IFN-I production than that of single deletions, suggesting that RSV exhibited a cooperative interaction between NS1 and NS2 to block the IFN-I singling pathway (Spann et al., 2004). Similar results were also observed in bovine RSV NS1 and NS2 proteins, which show cooperation in antagonizing IFN-I-mediated antiviral activity (Schlender et al., 2000). NS proteins exhibit multiple mechanisms to target different signals involved in the PRR signaling pathway to evade the IFN-I-mediated antiviral immune responses.

The importance of RIG-I in RSV recognition has been discussed above. However, NS1 and NS2 could interfere with the RIG-I-mediated antiviral signaling in various ways. In 293T cells, the direct interaction between NS2 and the CARD domains on RIG-I is confirmed by a coimmunoprecipitation (Co-IP) assay. This interaction prevents the signal transduction from RIG-I to downstream adaptor protein MAVS, which subsequently suppresses further induction of IFN-I and ISGs (Ling et al., 2009). Recently, a combined biochemical and structural analysis of NS2 protein confirmed again that NS2 could bind the CARD domain of both RIG-I and MDA5. It was revealed that the N terminus of NS2 is essential for binding an inactive form of RIG-I and MDA5, which subsequently prevented the transduction of downstream signaling and IFN-I production (Pei et al., 2021). During RSV infection in A549 cells, NS1 was visualized to be co-localized with MAVS in the mitochondria, and the direct interaction was further determined by Co-IP experiments (Boyapalle et al., 2012). Similarly, the interaction between NS1 and MAVS prevents signal transduction from RIG-I to MAVS without significantly affecting either RIG-I or MAVS expression. The tripartite motif-containing protein 25 (TRIM25) is an important E3-ubiquitin ligase responsible for delivering the K63-linked polyubiquitin chain to the CARDs domain in RIG-I to induce its oligomerization and activation. Evidence shows that the ectopic expression of NS1 could interact with TRIM25 and then interfere with the RIG-I ubiquitination to block the IFN-I signaling pathway (Ban et al., 2018). TRAF3 is essential for activating IRF3 and IRF7, which serves as the integration point of signals from both the RIG-I- and TLR-mediated IFN-I pathways. It is reported that the ectopic expression of either NS1 or NS2 reduces the protein level of TRAF3 in A549 cells, while only infection with NS1-deficient RSV, but not NS2-deficient virus increases the levels of TRAF3 compared to WT virus infection. Besides, the ectopic expression of NS1 but not NS2 decreases the levels of IKKε (inhibitor of IκB kinase ε), which is a Ser/Thr kinase of the IKK downstream of TRAF3. The further structure–activity studies demonstrated that the C-terminal 20 residues of NS1 were required to decrease IKKε (Swedan et al., 2009). These results suggest that NS1 can block the IFN-I signaling by reducing the presence of adaptors downstream RLRs. Moreover, NS1 and NS2 can also cooperatively block translocation of IRF3 to the nucleus, as shown by the increased nuclear presence in either NS1-deficient or NS2-deficient RSV-infected cells, which occurs by preventing the upstream phosphorylation of IRF3, or binding and sequestering IRF3 to prevent downstream association (Spann et al., 2005). Evidence from another study reveals a novel mechanism utilized by NS1 to impede the IFN-I signaling pathway. It is reported that NS1 does not affect RSV-induced phosphorylation and nuclear translocation of IRF3 but prevents its association to transcriptional coactivator CBP [cAMP response element-binding protein (CREB)-binding protein] and subsequently reduced binding of IRF3 to the IFN-β promoter in A549 cells (Ren et al., 2011).

RSV strains deficient in NS1 and NS2 expression show incomplete suppression of IFN-I production in human macrophage and epithelial cells, indicating that some other immunomodulatory proteins may help to the evasion of the early antiviral response. The N protein is essential for RSV assembly and replication as part of the nucleocapsid that functions as a template for replication and transcription by the viral polymerase complex. It is reported that the RSV N protein could co-localize with MDA5 in viral inclusion bodies (IBs) and antagonize the IFN-I response by interacting with MAVS (Lifland et al., 2012). Moreover, a recent study suggested that bovine RSV (bRSV) infection could also promote the formation of IBs-like structures induced by both viral N and P proteins. Interestingly, these organelles (IBs-like structures) can capture the p65 subunit of NF-κB, which further prevents its translocation to the nucleus (Jobe et al., 2020). It is a novel mechanism for viral antagonism of the IFN-I signaling pathway.

### RSV Subverts the TLRs Signaling Pathway

The glycoprotein protein (G) of RSV is responsible for the attachment of the virus particle to the target cell. It is expressed as membrane-anchored (mG) and secreted (sG) forms, containing a central fractalkine-like CX3C motif. It was demonstrated that the G protein was implicated in altering cytokine and chemokine expression in pulmonary leukocytes (Tripp, 2004). In the mouse infection model, the RSV F protein was reported to be an effector to trigger the production of IFN-β *via* interaction with TLR4 (Kurt-Jones et al., 2000; Haynes et al., 2001); while in monocyte-derived dendritic cells (mDCs), the soluble G protein was demonstrated to inhibit TLR3/4-mediated IFN-β production (Shingai et al., 2008). But the mechanism was not revealed in this study. The suppressor of cytokine signaling (SOCS) proteins family, especially SOCS1 and SOCS3 have elicited interest as negative regulators of IFN-I and pro-inflammatory cytokine signaling (Yoshimura et al., 2007). In macrophages and dendritic cells, both SOCS1 and SOCS3 were demonstrated to play an important regulatory role modulating TLR signaling (Takagi et al., 2004). Data from an RSV mutant virus lacking the G gene showed that the G protein has an important role in modulating SOCS1 and SOCS3, which subsequently downregulated the expression of IFN-I through the TLR signaling pathway (Oshansky et al., 2009).

### RSV Interferes With the JAK/STAT Signaling Pathway and Downstream ISGs

The antiviral activities of IFN-I are initiated by the engagement of IFNα/β to their cognate receptors IFNAR1/2 (IFNα receptors 1 and 2), which subsequently activate the downstream signaling cascades, namely JAK (Janus kinase)/STAT (signal transducer, and activation of transcription), resulting in the induction of various IFN-stimulated genes (ISGs). At present, more than 300 ISG proteins are identified to participate in the host’s antiviral immune responses. Multiple reports suggest that the NS proteins, singly or together, also affect the JAK/STAT signaling to block the production of ISGs, among which the STAT2 is shown to be the major target to be degraded caused by the NS proteins. An earlier study suggested RSV infection with a high inoculum at a multiplicity of infection (MOI) of 10 or ectopic expression of NS1 and NS2 in human epithelial cells A549 significantly decreased the protein level of STAT2 and fully inhibited IFN-β responsiveness (Lo et al., 2005). In contrast, a study from another group demonstrated that NS2 alone is sufficient and necessary for RSV to promote STAT2 degradation. During RSV infection in human tracheobronchial epithelial (hTBE) cells, the expression of NS2 under conditions could markedly decrease the protein levels of STAT2. Nonetheless, RSV infection with NS2 deletion did not affect STAT2 expression (Ramaswamy et al., 2006).

Moreover, when hTBE cells were pretreated with non-specific proteasome inhibitor MG-132, NS2 could not alter the expression of STAT2, indicating that NS2 mediated the degradation of STAT2 through a proteasome-dependent manner (Ramaswamy et al., 2006). But the complicated mechanism utilized by NS2 was still unclear in this research. Later, studies from the same group revealed that the C-terminal 10 residues were required for NS2 to decrease STAT2 (Swedan et al., 2009). Besides, the C-terminal DLNP tetrapeptide was also reported to be critical for NS2 to mediate the degradation of STAT2 *via* direct binding to the host microtubule-associated protein 1B (MAP1B) in the mitochondria. It was inferred that the bound of NS2 and MAP1B complex could serve as docking for mitochondrial receptors or recruit other host proteins to promote the destruction of STAT2 (Swedan et al., 2011). In addition to negatively regulating the TLRs signaling pathway to suppress IFN-I production, the SOCS proteins could also block the activation of the JAK/STAT signaling pathway *via* a negative feedback regulatory mechanism, of which SOCS1 and SOCS3 appear to be the most effective regulators (O’Shea et al., 2002; Akhtar and Benveniste, 2011). Mechanically, SOCS binds to JAK and inhibits the activity of its catalytic kinase reaction to block the JAK/STAT signal transduction pathway to inhibit STAT phosphorylation. It has been demonstrated that the NS1 protein can form a functional E3 ligase like SOCS, in which a putative elongin C and cullin 2 binding consensus sequences were identified by bioinformatic analysis (Elliott et al., 2007). The *in vitro* protein pulldown experiments also suggested that NS1 could act as a scaffold on which a multisubunit E3 ligase complex can be formed, which was essential for NS1/2-induced STAT2 degradation *via* a ubiquitin-dependent proteasomal mechanism (Elliott et al., 2007). Afterward, the NS1 was shown to induce the expression of SOCS1 in a RIG-I and TLR3-independent pathway, which further inhibit the phosphorylation of STAT2. It is a novel mechanism for NS1 to subvert the JAK/STAT pathway through an indirect way (Xu et al., 2014).

In addition, NS1 protein was also proved to inhibit the IFNAR synthesis through a viral replication-dependent manner. MicroRNAs (miRNAs) are small non-protein-coding RNA molecules of approximately 20 ~ 22 nucleotides encoded in the genome that bind to mRNA, leading to mRNA degradation or translational suppression. Viral infection, especially with RNA viruses, can usually cause the change of some cellular microRNA expression, potentially to the benefit of the virus. Data from the dual-luciferase reporter assay system demonstrated that during RSV replication in A549 cells, NS1 could upregulate the expression of miR-29a, which in turn targeted the mRNA of the *IFNAR* gene for degradation, resulting in the downregulation of protein translation of IFNAR. It is a novel mechanism for NS1 to subvert the JAK/STAT pathway through inducing the expression of miRNA as a negative regulator of IFNAR synthesis (Zhang et al., 2016).

The 2′-5′-oligoadenylate synthetases (OAS) are a family of ISGs characterized by their ability to synthesize 2′-5′-oligoadenylate and further induce RNA degradation. They consist of four OAS isoforms: OAS1, OAS2, OAS3, and OASL (2′-5′-oligoadenylate synthetases-like protein; Zhu et al., 2015). It was reported that OASL could inhibit viral replication during RSV infection. However, the NS1 protein seemed to possess the capacity to promote the degradation of OASL through a proteasome-dependent manner (Dhar et al., 2015). Moreover, the G protein also impedes the ISG15 expression in mouse lung epithelial (MLE)-15 cells, a type II pneumocyte cell line representing the distal bronchiolar and alveolar epithelium. ISG15 is an important ISG protein released from cells to mediate extracellular cytokine-like activities and exhibits a close link to IFN-I-mediated antiviral signal transduction. It has been shown that in MLE-15 cells, the infection of RSV with G gene deletion (RSVΔG) could stimulate significantly higher levels of ISG15 compared to that of the WT virus, indicating that the G protein plays a critical role in modifying the expression of ISG15 (Moore et al., 2008). Besides, the G protein could also mimic CX3C chemokine fractalkine (also named CX3CL1) due to the similar structure (Tripp et al., 2001). The bound of G protein to the CX3C receptor remarkably impairs the innate immune response to RSV infection. While the deletion of the CX3CL1 motif in G protein results in increased levels of IFN-I/III, indicating that the interruption of CX3C-CX3CR1 interaction by G protein greatly benefits RSV to evade IFN-I/III-mediated innate immune response (Chirkova et al., 2013).

## Concluding Remarks

It is more than 60 years since human RSV was first discovered in 1957 (Chanock et al., 1957), and RSV has become one of the most important pathogens causing respiratory disease worldwide, especially in young children. Globally, RSV causes approximately 60,000 deaths of hospitalized children younger than 5 years old each year (Shi et al., 2017). There is still no approved means for the effective prevention and treatment of RSV infection. Researchers have devoted themselves to studying the interaction between RSV and the host innate immune system in the past decades. The innate immune response plays an important role in defense against RSV infection. The elucidation of the recognition mechanism and viral innate immune evasion strategies caused by RSV contributes to designing novel drugs targeting potential regulatory molecules in the host innate immune pathway. Although the viral genome size is quite small and only encodes 11 viral proteins, RSV has evolved effective strategies to manipulate the host’s innate immune responses. In particular, the non-structural proteins NS1 and NS2 were suggested to degrade or sequester multiple signaling proteins that affect both IFN induction and IFN effector functions. The involved evasion mechanisms employed by NS1/2 are diverse, among which the proteasome-mediated direct degradation of target signals is most widely studied. In addition, NS proteins also indirectly affect the IFN-I response, such as inducing the expression of SOCSs and miRNAs to regulate the signaling pathway negatively in STATs and IFNARs levels, respectively. Intriguingly, the evasion strategies utilized by RSV are mainly concentrated on interfering with the pivotal signal molecules implicated in the RLRs pathway, such as receptors RIG-I and MDA5, adaptor protein MAVS, and transcription factors IRF3 and NF-κB.

However, the current knowledge regarding the NLRs, especially whether and how RSV blocks the NLRP3-mediated innate immune pathway, is still unknown. The present studies that characterize the role of NLRP3-mediated inflammasome are supposed to contribute mainly to RSV immunopathology and lung inflammation (Malinczak et al., 2021). NLRP3 inflammasome is suggested as a potential therapeutic target to attenuate severe RSV disease and limit childhood asthma development. In addition, the NF-κB induced pro-inflammatory cytokines expression is also considered to promote RSV-associated acute bronchiolitis (Zhang et al., 2021). A new understanding of the critical role of NLRP3 and NF-κB in triggering the antiviral innate immune response and aggravating the severity of the respiratory diseases during RSV infection is challenged. Therefore, additional studies on the interaction between RSV and host defense immune signaling are encouraged. These studies may provide novel antiviral therapeutic strategies to target viral components or cellular immune signals.

## Author Contributions

HZ, YO, and HL contributed ideas for the review and wrote the manuscript. KL and SH constructed the figures. HZ and YH edited and revised the manuscript. All authors contributed to the article and approved the submitted version.

## Funding

This work was supported by the Natural Science Foundation of Jiangxi Province (20202BAB216002), the Key Research Project of Gannan Medical University (ZD201906), the National Natural Science Foundation of China (82003043), and the training plan for academic and technical leaders of major disciplines in Jiangxi Province (20212BCJL23049).

## Conflict of Interest

The authors declare that the research was conducted in the absence of any commercial or financial relationships that could be construed as a potential conflict of interest.

## Publisher’s Note

All claims expressed in this article are solely those of the authors and do not necessarily represent those of their affiliated organizations, or those of the publisher, the editors and the reviewers. Any product that may be evaluated in this article, or claim that may be made by its manufacturer, is not guaranteed or endorsed by the publisher.
